# Raman Spectroscopy as a PAT-Tool for Film-Coating Processes: In-Line Predictions Using one PLS Model for Different Cores

**DOI:** 10.3390/pharmaceutics12090796

**Published:** 2020-08-23

**Authors:** Juliana Radtke, Hubertus Rehbaum, Peter Kleinebudde

**Affiliations:** 1 Institute of Pharmaceutics and Biopharmaceutics, Heinrich Heine University, 40225 Duesseldorf, Germany; juliana.radtke@hhu.de; 2 Dr. Rehbaum Technology Consulting GmbH, 14195 Berlin, Germany; h.rehbaum@rehbaum.info

**Keywords:** Raman spectroscopy, process analytical technology (PAT), coating, partial least squares, chemometrics

## Abstract

Although Raman spectroscopy has been described as a potential process analytical technique for tablet coating, it has rarely been transferred from academic studies to commercial manufacturing applications. The reasons for this are probably not only the high level of process understanding and experience with multivariate data analysis required, but also the product-dependent elaborate model-building. Hence, this study represents a feasibility study to investigate, whether subtraction of core spectra is a suitable approach to generate versatile models for one specific coating that can be applied on a multitude of different tablet cores. Raman spectroscopy was used to predict the application of coatings on three different tablet cores using PLS regression. The obtained spectra were preprocessed, and differential spectra were calculated by subtraction of the core spectrum from each inline spectrum. Normalization ensured comparability between the spectral data of the different cores. It was shown that in general it is possible to build models for a specific coating suspension that can predict the application of this suspension on different cores. In the presence of a strong Raman marker (TiO_2_), promising results were obtained. Without the presence of a strong Raman marker this modeling approach is to be considered critical.

## 1. Introduction

Tablet film coating is a common pharmaceutical process in which functional, non-functional and active coatings can be applied onto tablet cores [1]. To ensure the safety and efficacy of the product, a consistent quality of the coating is required. A critical quality attribute of the film coating is the film thickness. On one hand, for non-functional and enteric coatings a minimum film thickness is necessary to ensure the functionality of the coating. On the other hand, increased film thickness can delay the drug release. In sustained release formulations, the release of the drug is determined by the film thickness. In active coatings, the film thickness determines the amount i.e., dosage strength of the active pharmaceutical ingredient (API). In coating processes, the quality of the product can be influenced by many process parameters and their interactions, i.e., inlet and outlet air temperature, spray rate, pan speed, negative pressure of the drum, etc. The monitoring of the process by using process analytical technology (PAT) can help to detect deviations and problems during the process and in consequence increase the quality of the final product [2]. The importance of the implementation of PAT was emphasized in a guidance by the Food and Drug Administration (FDA) in 2004 [3]. The FDA describes PAT as “a system for designing, analyzing and controlling manufacturing through timely measurements (i.e., during processing) of critical quality and performance attributes of raw and in-process materials and processes, with the goal of ensuring final product quality”. PAT tools are divided into four different categories: “multivariate tools for design, data acquisition and analysis; process analyzers; process control tools and Continuous improvement and knowledge management tools”. Some or all of these tools should be combined to assure the quality of an entire manufacturing process. In inline measurements the sample is not removed from the process and thus a suitable process analyzer should be nondestructive and not require any sample preparation. In addition, immediate measurements are necessary. One such technology is Raman spectroscopy [4], meeting all given requirements. In addition, it allows measurements in the presence of water, as water only results in neglectable Raman scattering. This makes Raman spectroscopy a suitable PAT tool even for aqueous film coatings. Consequently, in the last years Raman spectroscopy has been successfully implemented as a PAT tool for active coatings [5,6], various polymers and colored coatings [7]. Transferring it to similar processes, it was even used to monitor a multilayered film coating on pellets [8].

Besides the already mentioned Raman spectroscopy [5,6,7,9,10,11,12], further PAT tools were developed to monitor the coating thickness during the coating process. To be mentioned are near infrared spectroscopy [13,14,15,16,17,18], terahertz-pulsed technology [10,19,20,21,22,23,24], optical coherence tomography [22,25,26,27] and visual imaging techniques [1,28,29,30]. Most of these process analyzers generate large volumes of multidimensional data. Information on the process can be extracted from these data by using univariate or multivariate modeling approaches [31]. In univariate data analysis, the identified variables are considered individually. In the application to Raman spectroscopy this refers to evaluating only a single Raman band. However, the derived data usually are complex, requiring multivariate approaches to extract process relevant information from the large data sets. Both univariate and multivariate approaches for the analysis of Raman spectra as a PAT tool for coating processes were applied and compared in different publications. Barimani and Kleinebudde compared the feasibility of science-based calibration (SBC), PLS-regression (PLSR) and univariate data analysis (UVDA) in end-point determination of coating processes using Raman spectroscopy [12]. In this study, SBC showed the highest prediction errors, while the errors which were obtained by UVDA were comparable to errors resulting from multivariate data analysis methods (MVDA). This was explained by the presence of titanium dioxide (TiO_2_), a highly Raman-active ingredient providing a signal increase over time. Regarding commonly used MVDA approaches, PLSR is to be named as the most recommended multivariate approach [32], as well as multivariate curve resolution–alternating least squares (MCR–ALS). In literature, comparative studies on the predictive ability of PLSR and MCR–ALS led to different results. Hisazumi and Kleinebudde [8] described a prediction superiority of MCR–ALS for multilayered coating layers in a pellet-coating process. However, in a three-layer tablet coating process, PLSR was shown to be more suitable for the prediction of all layers, while MCR–ALS failed in predicting the application of the third layer [33]. The comparison of both MVDA methods by Azzouz and Tauler in UV and NIR spectrophotometric measurements resulted in prediction errors in the same range [34]. A big advantage of PLSR is its ability to handle noisy, collinear and incomplete data in both dimensions [35].

From the viewpoint of model building, for quantitative UVDA analysis the selection of the wavenumber is the most critical step, as the modeling procedure relies only on this wavenumber. The most critical step in PLSR is the selection of the number of factors. The correlation between X- and Y data will be insufficient, if the number of chosen factors is too low. Choosing the number of factors too high, can lead to an “overfitting” and instability with a decreasing predictive ability of the model. Raw data—which are derived from the process analyzer—may often not be optimal for analysis. Interferences such as background effects, baseline shifts, and varying measurement conditions can affect the absolute intensity of the derived spectral data. The noise that is caused by such effects can be minimized by using different preprocessing methods. Another critical step in spectral MVDA is the selection of the spectral range that is included into the model and the exclusion of outliers. After the implementation of a chemometric model it should be reassessed regularly to ensure a consistent level of acceptable prediction performance [32]. Taking the above-mentioned challenges of the model building procedure into account, it should only be performed by qualified scientists with knowledge about the process and a deep understanding of MVDA.

The suitability of Raman spectroscopy as an inline tool during film coating was shown in the literature. As the modeling procedure is complex and time-consuming, the aim of this study was to verify, if it is possible to predict the application of the same coating dispersion on different cores using the same model. This could reduce the effort of model building procedures and increase the applicability of Raman spectroscopy in coating processes. In the best-case scenario, core-independent models for different coating preparations can be provided and used by pharmaceutical companies for a multitude of products. This study is meant to be a first basic feasibility study.

## 2. Materials and Methods

### 2.1. Tablet Cores

Coating suspensions were applied on three different biconvex tablet cores: acetylsalicylic acid (ASA) cores, diclofenac cores and placebo cores. ASA cores were produced via direct compression using a Korsch XL 200 tablet press (Korsch AG, Berlin, Germany). They consisted of 62.1% ASA, 6.9% corn starch, 30% microcrystalline cellulose (Vivapur^®^ 102, J. Rettenmaier & Söhne GmbH + Co KG, Rosenberg, Germany) and 1% sodium stearyl fumarate (Pruv^®^, J. Rettenmaier & Söhne GmbH + Co KG, Germany). The cores had an average mass of 239.6 mg, a diameter of 8.0 mm, a 4.2-mm height and a 2.8-mm band height. Diclofenac cores were produced via direct compression in a Korsch XM 12 (Korsch AG, Germany), containing 12.5% diclofenac sodium, 36.5% alpha–lactose monohydrate (FlowLac^®^ 100, Molkerei Meggle Wasserburg GmbH & Co. kG, Wasserburg am Inn, Germany), 50% microcrystalline cellulose (Sanaq 102, Pharmatrans-Sanaq AG, Allschwil, Switzerland) and 1% magnesium stearate (Parteck LUB MST, Merck KGaA, Darmstadt, Germany). These cores were produced with an average mass of 205.6 mg, a diameter of 8.0 mm, a tablet height of 4.02 mm and a 2.02-mm band height. Placebo cores were provided by L.B. Bohle Maschinen + Verfahren GmbH. They consisted of 99% dicalcium phosphate (DI-CAFOS^®^ 150, Chemische Fabrik Budenheim KG, Budenheim, Germany) and 1% magnesium stearate. The placebo cores had an average mass of 252.4 mg, a diameter of 8.1 mm, a tablet height of 3.42-mm and a band height of 2.05 mm.

### 2.2. Coating Suspensions

Experiments were performed using two different coating suspensions. One suspension was an enteric coating, while the other was an immediate release coating. The first coating suspension was an enteric ready-to-use coating based on a copolymer of methacrylic acid and ethyl acrylate. The suspension contained 20% AquaPolish^®^ (AP) P white 712.02 E (Biogrund GmbH, Hünstetten, Germany), 2% propylene glycol (Cesar & Lorentz GmbH, Hilden, Germany) and 78% demineralized water. AquaPolish^®^ P white 712.02 E is a one-step film coating system containing acrylic acid copolymer and TiO_2_ in the anatase modification. The second coating suspension consisted of 15% AquaPolish^®^ P white 014.117 (Biogrund GmbH, Hünstetten, Germany) and 85% demineralized water. AquaPolish^®^ P white 014.177 is a TiO_2_-free white HPMC/HPC-based ready-to-use mixture. Calcium carbonate and dicalcium phosphate are contained as white pigments. Both coating suspensions were permanently stirred during the coating process.

### 2.3. Coating Process

For each coating dispersion, six batches were coated, resulting in a total of twelve batches. Tablet batch size for each coating run was 3800 g. The six batches per coating dispersion each consisted of two batches of ASA, diclofenac and placebo cores. One or more batches, further referred to as calibration batches, were used for model building, and the resulting model was then applied to the second batch, the test batch. The cores were coated in a BFC 5 pan coater with a 5-L drum (L.B. Bohle Maschinen + Verfahren GmbH, Ennigerloh, Germany). Coating dispersion was applied using two 1.0-mm nozzles (Düsen-Schlick GmbH, Untersiemau, Germany) that were installed at a distance of 10 cm from tablet bed. The process parameters used for the coating runs are listed in Table 1. All parameters were kept constant for the six batches of each coating dispersion. For the enteric coating, the aimed mass gain was 8%—which corresponds to a sprayed coating mass of 1373 g. For the immediate release coating, the aimed mass gain was 5%—which corresponds to a sprayed coating mass of 1330 g. The applied coating mass was monitored by weight during the coating run.

### 2.4. Coating Thickness Determination

To calculate the resulting endpoint coating thickness, the volume of the coating on the cores and the density of this coating was required. To assess the volume, the total tablet surface was determined by measuring tablet dimensions using a caliper (Mitutoyo^®^, Kawasaki, Japan) and a tablet tester (Smart Test 50, Dr. Schleuniger Pharmatron, Thun, Switzerland). Then, the volume of the film was calculated from the predicted coating mass and total tablet surface. To determine the density of the film, films were produced using the solvent casting method on a Coatmaster 510 (Erichsen GmbH & Co. KG, Hemer, Germany). The plate was tempered to 33 °C for the enteric coating dispersion and to 41 °C for the immediate release coating dispersion. The suspensions were casted with a coating knife, set to a gap width of 800 µm. The drying time was between 45 and 60 min. After the film forming was completed, the density of the film was measured by gas pycnometry (AccuPyc 1330, Micrometics Instrument Corp., Norcross, GA, USA). During gas pycnometry measurements, the temperature was set constant to 25 ± 0.1 °C. The resulting coating thickness was calculated for the endpoint which was predicted with the inline model APD1 and APD2.

### 2.5. Raman Spectroscopy

Raman spectra were collected inline using a Raman RXN2 spectrometer (Kaiser Optical Systems, Inc., Ann Arbor, MI, USA). To allow for the measurement of a larger sample area, a PhAT probe was installed. This noncontact optic device forms a spot with a diameter of 6 mm, i.e., a spot area of 28.3 mm^2^. The diode laser emits photons at 785 nm and has a maximum power of 400 mW. To allow for inline measurements, the PhAT probe was installed through the front door of the coater and positioned in a way that a constant measuring spot was formed on the moving tablet bed. The optic was permanently dedusted with compressed air during the coating process. The probe was installed in a measuring distance of 21 cm from the tablet bed. The iC Raman™ 4.1 software package (Kaiser Optical Systems, Inc., Ann Arbor, MI, USA) was used for data acquisition. One spectrum was measured every ten seconds throughout the entire process time. The exposure time was set to 2 s for both coating suspensions. A light cover was used for all measurements to exclude interference from foreign light sources, e.g., the room lightning.

### 2.6. Data Selection for Model Building

Core-specific calibration models were built using the calibration data sets of all three core types. Model building was carried out separately for both coating suspensions. To determine the applicability of the models for other cores, models were tested by predicting the application of the respective coating dispersion for the test batch of the same and all other cores. For the enteric coating, a fourth model including all calibration data sets from the three different cores was built. This model then was used to predict the three test data sets. Table 2 provides an overview of the data that were used for model building and testing.

### 2.7. Data Analysis Methods

#### 2.7.1. Data Pretreatment

The raw spectra were preprocessed using standard normal variate (SNV) and Savitzky–Golay smoothing (2nd derivative, window size of 21). Varying settings as well as further preprocessing procedures like baseline correction were tested, but did not improve the models further. Preprocessing was performed in Matlab^®^ R2018b (The MathWorks, Inc., Natick, MA, USA). The preprocessed range of wavenumbers depended both on the applied coating suspension and the used cores. It was chosen manually for each model with regard to the model performance parameters. To ensure an optimal spectral range, models were built and tested with different spectral ranges. The range of wavenumbers of the spectral data returning the smallest root mean square error of prediction (RMSEP) was chosen for final model-building. A moving average with a window size of six was carried out for all inline-measured spectra. This corresponds to a process time of 60 s.

#### 2.7.2. Differential Spectra

To reduce the core-specific information and enhance the coating-specific spectral changes during the coating process, a mean core spectrum was continuously subtracted from the inline measured spectra. To calculate the mean core spectrum, eight core spectra were measured inline at the end of the warmup phase, just before starting the spraying phase. These spectra were each preprocessed using SNV before calculating the mean spectrum. The derived mean spectrum was subsequently subtracted from every inline measured and SNV-filtered spectrum.

#### 2.7.3. Spectral Normalization

**Figure 1:**
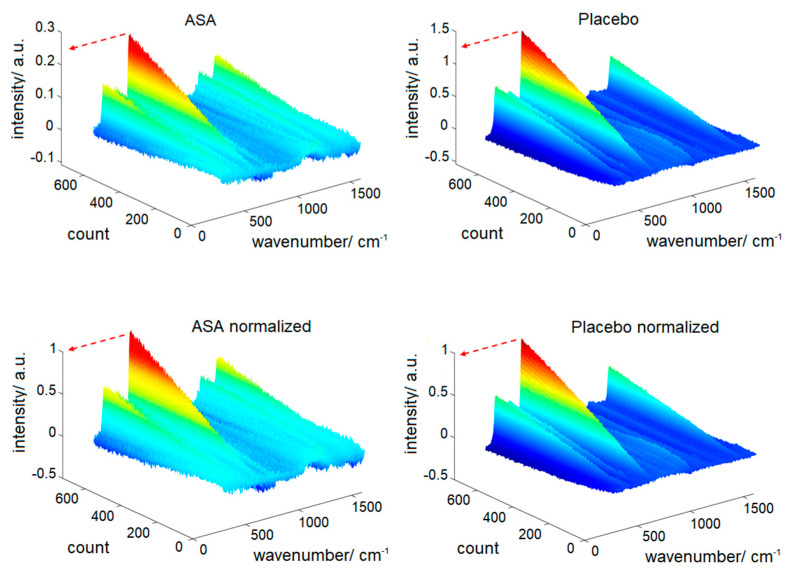
Standard normal variate (SNV)-preprocessed differential spectra; (**top**) before and (**bottom**) after normalization.

Depending on the Raman intensity of the core material, the obtained differential spectra showed distinct absolute intensities. To make comparability of the varying differential spectra possible, a normalization method was required. Hence, all differential spectra were normalized by the SNV maximum of the last inline measured spectrum. Using this normalization, the largest peak increases along the process time to reach 1 by the end of the process (Figure 1). This normalization method is labeled as “max” in the following. For the enteric coating containing TiO_2_ a second normalization approach was tested. Here, the spectra were normalized using the mean intensity of the three TiO_2_ peaks at the end of the process, which can be found at 398, 516 and 640 cm^−1^. This normalization method is abbreviated as “max_3_”. The three peak intensities were extracted from the individual spectral datasets at the end of the process, consequently representing their maximum values. As this approach is not suitable for inline measurements, normalization of the test data that were predicted by models APD1 and APD2 was performed using the maxima that were determined from the calibration data set.

#### 2.7.4. PLS Regression

PLS regression models were created in Matlab^®^ R2018b, with the calibration models built by correlating the preprocessed spectra to the amount of applied coating mass. The spectral data of the entire coating run were considered for model building. Strong outliers were identified by using the Hotellings T^2^-test and excluded before final model building. Outliers frequently occurred at the beginning of the process, as the process was not yet stable. Outliers were excluded with regard to the prediction ability of the model. The number of factors and the spectral range contributing to the model was chosen to optimize for the smallest RMSEP. A smaller or higher number of factors resulted in higher RMSEPs, caused either by underfitting or overfitting of the data. The optimal parameters were determined individually for each model.

#### 2.7.5. Model Performance Parameters

To estimate the quality of the calibration model, the coefficient of determination (R^2^), the root mean square error of calibration (RMSEC) and the standard error of calibration (SEC) were calculated. The developed models were used to predict the applied coating mass of a new test set. Afterwards, the RMSEP was calculated to evaluate the predictive performance ability of the models. RMSEC, RMSEP and SEC are provided as a percentage of the applied coating mass.

## 3. Results and Discussion

### 3.1. Comparison of the Different Core Spectra

**Figure 2:**
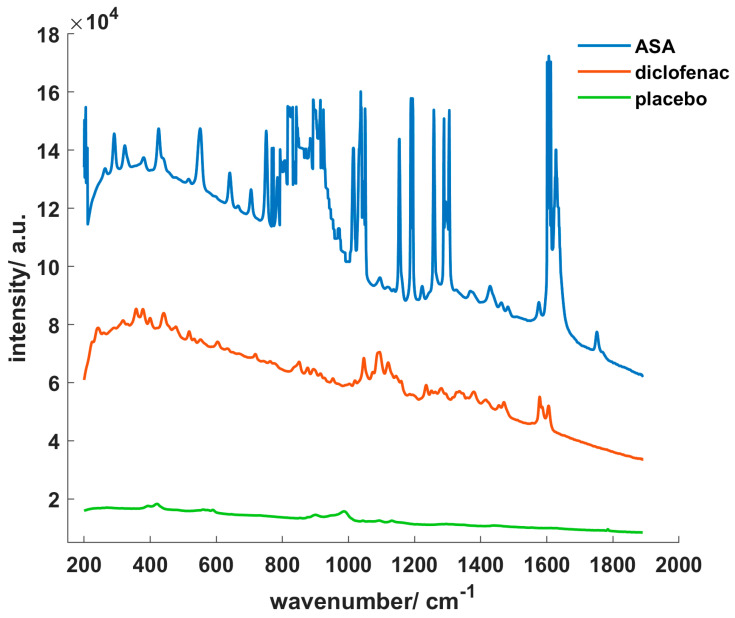
Raman spectra of acetylsalicylic acid (ASA), diclofenac and placebo cores.

Though all core spectra were measured with the same exposure time of 2 s, the obtained raw spectra of the three cores differed clearly, as is displayed in Figure 2. ASA cores with the highest API content exhibited the highest absolute Raman intensity and very strong peaks. The characteristic ASA double peak occurred at a wavenumber around 1605 cm^−1^, other characteristic peaks were located between 990 and 1340 cm^−1^. In comparison, diclofenac cores showed an overall lower intensity. Again, while many peaks are visible, those were less distinctive. Characteristic diclofenac peaks occurred at 441, 1046, 1578 and 1605 cm^−1^. As expected, placebo cores demonstrated the lowest base intensity. Only few peaks were present with a weak intensity.

### 3.2. Comparison of the Different Coating Suspension Spectra

**Figure 3:**
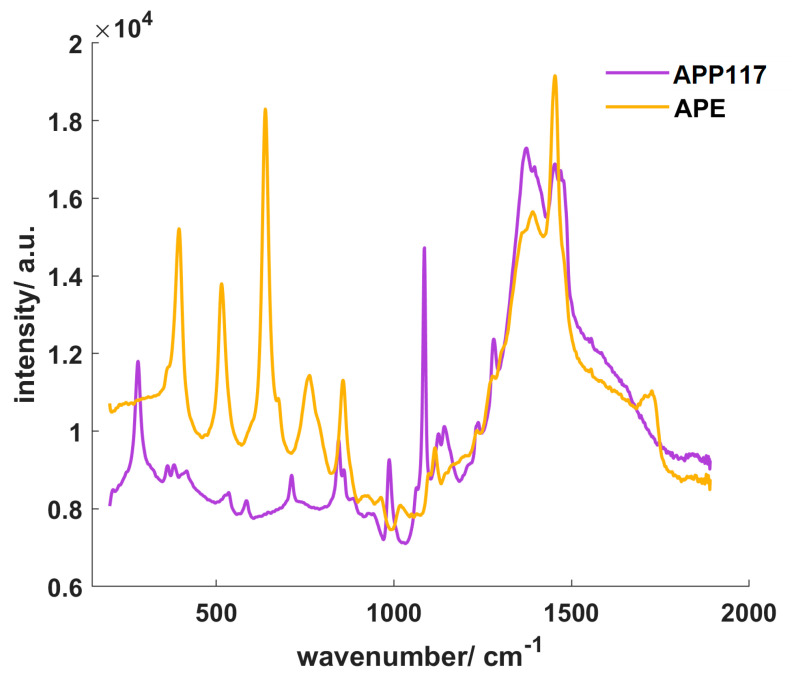
Raman spectra of the different coating layers, APE: AquaPolish^®^ P white 712.02 E; APP117: AquaPolish^®^ P white 014.117.

The spectra of the casted films were again measured with the same exposure time (2 s). AquaPolish^®^ P white 712.02 E (APE) showed higher overall Raman intensities at most wavenumbers (Figure 3). As the enteric coating contains TiO_2_ in the anatase modification, the three most characteristic TiO_2_ peaks of this modification are present at 398, 516 and 640 cm^−1^. In comparison, AquaPolish^®^ P white 014.117 showed lower Raman intensities with characteristic CaCO_3_ peaks at 280, 712 and 1086 cm^−1^.

### 3.3. PLSR Models for TiO_2_-Containing Coatings on Different Cores

#### 3.3.1. PLSR Calibration and Prediction Model Performance

In Table 3 the calibration model performance parameters of the models are provided that were optimized towards the smallest prediction errors. All errors listed in the table are given as a percentage of the applied coating mass. TiO_2_ showed a strong intensity increase during the coating run, which enabled building reliable models. In this study, the anatase modification was used—which shows three characteristic peaks at 398, 516 and 640 cm^−1^. A number of two or three factors was chosen and resulted in an R^2^ higher than 0.999 for all models. The models returned calibration errors <0.7%. As an exception, ASA models—which were built with smaller wavenumber ranges (<1000 cm^−1^)—demonstrated higher calibration errors. This may be caused by the high intensity of the core spectra. ASA cores showed the strongest signal of all cores—which led to a smaller intensity difference between the increasing TiO_2_ peak and the core peak—as well as a higher remaining noise after preprocessing and normalization procedure.

Overall, it can be seen that—not surprisingly—applying models that were built on the same core as the test sets led to the best predictions with lowest RMSEPs (A1.1/2.1, P1.2/2.2, D1.3/2.3). A2.3 and D1.2 are exceptions from this trend, but also indicate a strong dependency on the normalization method and, therefore, are probably not sufficiently robust, as will be discussed in in Section 3.3.2. Furthermore, coatings on cores with low or moderate innate Raman activity, i.e., diclofenac and placebo, were predicted satisfactorily by all models. Though the prediction errors were lowest for models based on placebo and diclofenac cores (P1.2, P2.2, P1.3, P2.3, D1.2, D2.2, D1.3, D2.3), also those based on ASA cores (A1.1–A2.3) were in an acceptable range.

However, predicting the coating on ASA cores that demonstrated a high Raman activity was challenging. When predictions were made using placebo models (P1.1/2.1), the resulting RMSEP depended strongly on the applied normalization method. While for the max_3_ method an unacceptably high error of 4.97% was obtained, the other method allowed precise predictions with an RMSEP of 1.03%. The models built on diclofenac cores did not allow predictions with acceptable errors at all, regardless of the applied normalization method. Here the lowest RMSEP which obtained was 3.32%.

#### 3.3.2. Comparison of the Different Normalization Methods

From the obtained data, no consistent effect of the normalization method was found if the same cores are used as calibration data set for model building and as the predicted test data set (A1.1/A2.1, P1.2/P2.2, D3.1/D3.2). For ASA cores, both normalization methods led to a RMSEP of 1.45%. For placebo and diclofenac cores RMSEPs of 0.94% and 1.49% were observed.

Concerning the models which were built with the ASA calibration data set, the max_3_ normalization method led to better prediction results for the other cores, compared to the max normalization method. Using the max normalization method, the application of coating mass was predicted with a prediction error of 2.08% on placebo cores and 2.86% on diclofenac cores. The max_3_ normalization method resulting in a RMSEP of 1.26% for the placebo cores and 1.65% for the diclofenac cores. In contrast, for placebo models the max normalization method provided smaller errors when predicting the other cores. Models that were built with spectra after max_3_ normalization showed an RMSEP of 4.97% for the ASA cores and 3.71% for the diclofenac cores, whereas the max normalization method led to smaller prediction errors with 1.03% for the ASA cores and 1.17% for the diclofenac cores. The max normalization method was also superior for the predictive ability of the diclofenac models. It allowed for a RMSEP of 3.32% for the ASA cores and 0.76% for the placebo cores, compared to errors of 5.71% (ASA) and 2.58% (placebo) which were observed by using the second normalization method.

One may assume that the max_3_ is especially suitable for spectra with high intensities and sharp peaks, as observed for ASA, while the max approach is superior for spectra of low intensity. However, a judicious conclusion on the best normalization technique would also focus on robustness and repeatability and is hence not yet possible based on the presented data. Probably though, highest robustness is to be expected for normalization techniques that take into account as much information as possible—in the best case in a multivariate way.

### 3.4. PLSR Models for Inline Predictions of TiO_2_-Containing Coatings

#### 3.4.1. PLSR Calibration Model Performance

As outlined before, normalization of the spectra was a prerequisite to the presented approach. It was performed using spectral information from the end point of the coating experiments. For the desired real time implementation however, max and max_3_ must be known from the beginning and thus cannot be determined at the end of the process. For this part of the study, max/max_3_ were extracted from the final spectra of the calibration experiments and applied to test data. With these values for max/max_3_, different preprocessing methods, the subtraction of the core spectrum, and the normalization procedure can be applied to the spectral data, hence allowing for predictions to be performed in real time. As shown in Table 4, the models which were built with the normalized differential spectra of the calibration data sets of all cores showed a smaller R^2^ compared with models shown in Table 4. However, R^2^ values higher than 0.99 were achieved and the calibration errors were in the same range as for the models shown before. A number of two factors was sufficient for both models.

#### 3.4.2. PLSR Prediction Model Performance

**Figure 4:**
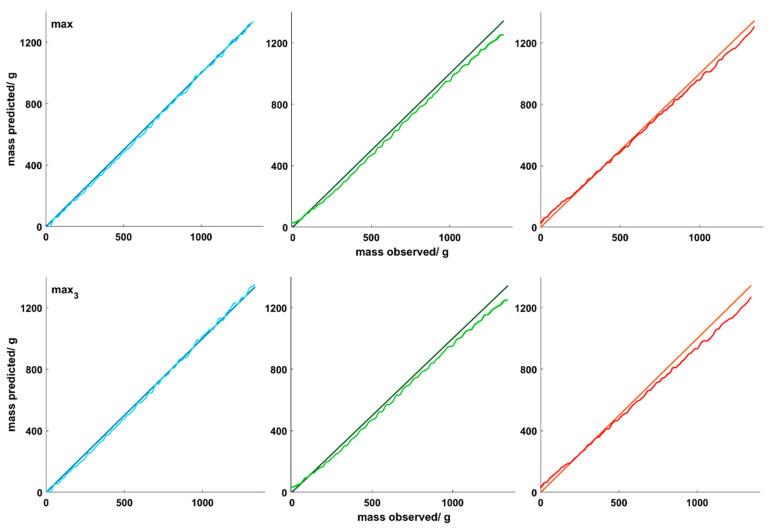
Observed versus predicted coating masses of (blue) ASA, (yellow) placebo and (red) diclofenac cores using the two different normalization methods.

The prediction errors for the applied coating mass on all three cores are reported in Table 4. The application of coating mass on the ASA cores was predicted with small errors of 0.79% for the first normalization method and 0.59% for the second normalization method. Considering the endpoint prediction, for the ASA cores the first normalization method resulted in a coating thickness of 75.1 µm and the second method in a thickness of 75.9 µm. As a true coating thickness of 75.1 µm was calculated, both models very precisely predict the applied coating mass on the ASA cores, although model APD1 may suggest a slightly higher precision compared to the APD2 model. For the placebo and diclofenac cores, higher prediction errors were obtained. Dividing all preprocessed spectra by the maximum resulted in an error of 2.46% for the placebo cores and 2.16% for the diclofenac cores. Figure 4 depicts that for both cores the coating mass is underestimated especially at the end of the process. Comparable results were obtained with both normalization methods. The highest deviations in predicting the endpoint coating thickness were obtained for the placebo cores. According to calculations, placebo cores showed an endpoint thickness of 87.6 µm. Using model APD1 resulted in an endpoint thickness of 81.5 µm, while model APD2 predicted a thickness of 81.3. With a deviation of 6.3 µm, model APD2 led to the poorest endpoint coating thickness prediction. Diclofenac cores had a calculated endpoint thickness of 71.6 µm. With the APD1 and APD2 model a thickness of 69.6 µm and 67.68 µm were predicted, respectively. Regarding the endpoint coating thickness, the max normalization method resulted in better predictions for all cores. For the placebo and diclofenac cores, the underestimation of the applied coating mass in the second half of the process caused unacceptable deviations from the predicted and the observed coating thickness. Inline process control would in this case result in the application of a too high coating mass. We are confident that the applicability of the presented approach can be extended to those cores by the optimization of the normalization method and probably further preprocessing of the recorded spectra.

### 3.5. PLSR Model for TiO_2_ Free Coatings on Different Cores

#### 3.5.1. PLSR Calibration Model Performance

Calibration model performance parameters are reported in Table 5. As the most characteristic peak of this coating emerges at 1086 cm^−1^, the model ranges differ from the ranges of the TiO_2_-coating models. The models are mostly based on higher wavenumbers. The modeling procedure for the CaCO_3_ containing coating was more challenging compared to the coating containing TiO_2_ (anatase). This is reflected in higher calibration errors. Especially for ASA and diclofenac cores—which showed distinctive peaks and overall higher intensities—this is equally reflected in higher calibration errors. As shown in Section 3.1, the ASA cores exhibited a high Raman activity, which caused the ASA peaks to cover most of the CaCO_3_ signal. Consequently, no suitable models were obtained by the ASA calibration set to predict the applied coating mass on placebo and diclofenac cores. The prediction model for the ASA cores returned an RMSEC of 1.08% and 4 factors were needed to obtain a R^2^ > 0.999. The model built using the spectra coating the placebo cores, led to R^2^ > 0.999. The optimal number of factors depended on the spectral range and was between 2 and 4 factors. Again, comparable higher RMSEC values were obtained except for model P3.3 which contained 4 factors and showed a small RMSEC of 0.06%. By using the spectra of the diclofenac core coating process, a R^2^ higher 0.999 was only achieved using 4 factors and a range between 900 and 1560 cm^−1^. For the other models, a R^2^ > 0.998 was received.

#### 3.5.2. PLSR Prediction Model Performance

As demonstrated in Table 5 the challenging model building procedure resulted in higher prediction errors. The applied coating mass on ASA cores was predicted with a minimum prediction error of 2.65% by the placebo model and a maximum error of 4.13% using the diclofenac model. The placebo cores showed the lowest Raman activity. Here, the growing CaCO_3_ peak was clearly visible which resulted in a small RMSEP of 1.16% using the placebo model (P3.1) and an acceptable RMSEP of 2.21% using the diclofenac model. The increase of CaCO_3_ can also be observed in the spectral data of the diclofenac cores. However, in comparison with the placebo cores, higher prediction errors of 2.14% (D3.3) and 2.71 (P3.3) were obtained.

**Figure 5:**
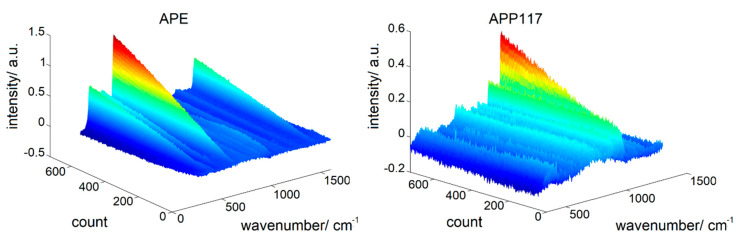
SNV-preprocessed differential spectra during the application of APE and APP117 on the placebo cores.

The higher prediction errors of the PLS-models of APP117 can be explained by considering the obtained differential spectra during the application of APP117 and APE. In Figure 5 the non-normalized differential spectra during the application of both layers on the placebo cores are shown. The most dominant TiO_2_-peak grows constantly during the coating process and the differential spectra demonstrate only slight noise in comparison to the differential spectra of APP117. Here, the spectra show a higher noise and the intensity of the most dominant CaCO_3_ peak is measured with fluctuating intensities. In addition, the TiO_2_ peak grows from an intensity of −0.01 a.u. to 1.24 a.u., while the CaCO_3_ peak grows from −0.005 a.u. to 0.453. This resulted in higher differences in the intensity of the obtained spectra during the application of the TiO_2_ containing coating.

## 4. Conclusions

The need for elaborate model building and optimization is a significant burden for the establishment of Raman spectroscopy as a commercially viable and broadly used process analytical technology. A new approach, the generation of models valid for specific coatings on a variety of different tablet cores by subtraction of core spectra, was demonstrated to be in principle suitable. The application of a TiO_2_-containing coating on three different tablet cores was successfully predicted with small prediction errors using models that were based only on the spectra of one of these cores. However, it was not possible to predict the applied coating mass on the ASA cores using the diclofenac model. Considering the two different normalization methods that were applied to the differential spectra, there was no clear superiority of one over the other. A more robust normalization method is needed, especially for a real inline implementation of this approach. If the values which were used for normalization of the test data set were based on the spectra of the calibration data set, reliable predictions were only achieved for the ASA cores. For the diclofenac and placebo cores, this resulted in an underestimation of the applied coating mass.

The prediction of the CaCO_3_-containing coating was more challenging using this approach. Higher prediction errors were observed for all cores and the models failed predicting the application of the coating mass on ASA cores. Hence, for the combination of cores with high Raman intensity and coatings with low Raman intensity, the model building is challenging, and no reliable predictions can so far be obtained.

In conclusion, it is in general possible to build PLS prediction models for a specific coating preparation that can be used to control the application of this preparation on different cores. Though this approach requires increased efforts during initial model development, it should be able to simplify model building significantly over longer terms. In the presence of TiO_2_, promising results were obtained, making the authors optimistic that this approach can be applicable for a high number of TiO_2_ containing coatings. For TiO_2_-free coatings—especially in the presence of highly Raman active ingredients in the cores—it is questionable if this approach is applicable.

## Figures and Tables

**Table 1:** Coating process parameters.

Coating Dispersion	Pan Speed/rpm	Spray Rate/g/min	Inlet Air Volume/m^3^/h	Exhaust Air Temperature/°C	Inlet Air Temperature/°C
AP P white 712.02 E	16	11–12	100	33	50
AP P white 014.117	16	11–12	100	41	60

**Table 2:** Data selection for model building and testing for both coating suspensions.

Coating	Calibration Data Set	Prediction Data Set	Normalization	Model	Normalization	Model
AP P white 712.02 E	ASA batch 1	ASA batch 2	max	A1.1	max_3_	A2.1
		placebo batch 2		A1.2		A2.2
		diclofenac batch 2		A1.3		A2.3
	placebo batch 1	ASA batch 2	max	P1.1	max_3_	P2.1
		placebo batch 2		P1.2		P2.2
		diclofenac batch 2		P1.3		P2.3
	diclofenac batch 1	ASA batch 2	max	D1.1	max_3_	D2.1
		placebo batch 2		D1.2		D2.2
		diclofenac batch 2		D1.3		D2.3
	ASA, placebo, diclofenac batch 1	ASA, placebo, diclofenac batch 2	max	APD1	max_3_	APD2
AP P white 014.117	ASA batch 3	ASA batch 4	max	A3.1	-	
		placebo batch 4		A3.2		
		diclofenac batch 4		A3.3		
	placebo batch 3	ASA batch 4	max	P3.1		
		placebo batch 4		P3.2		
		diclofenac batch 4		P3.3		
	diclofenac batch 3	ASA batch 4	max	D3.1		
		placebo batch 4		D3.2		
		diclofenac batch 4		D3.3		

**Table 3:** PLS-regression (PLSR) calibration and prediction model performance parameters of the TiO_2_-containing coating for ASA, placebo and diclofenac cores.

Cores	Model	Prediction	Normalization	Range [cm^−1^]	Factors	R^2^	RMSEC/%	SEC/%	RMSEP/%
ASA	A1.1	ASA	max	340–1400	3	0.9990	0.44	0.05	1.45
	A2.1		max_3_	340–1400	3	0.9998	0.42	0.05	1.45
	A1.2	placebo	max	340–900	2	0.9992	0.81	0.09	2.08
	A2.2		max_3_	340–1000	2	0.9993	0.77	0.09	1.26
	A1.3	diclofenac	max	340–700	2	0.9992	0.81	0.09	2.86
	A2.3		max_3_	340–900	2	0.9992	0.81	0.09	0.65
placebo	P1.1	ASA	max	340–1500	2	0.9996	0.58	0.07	1.03
	P2.1		max_3_	340–900	3	0.9996	0.54	0.07	4.97
	P1.2	placebo	max	340–1300	3	0.9996	0.54	0.06	0.94
	P2.2		max_3_	340–1300	3	0.9996	0.54	0.06	0.94
	P1.3	diclofenac	max	340–1500	2	0.9996	0.58	0.07	1.17
	P2.3		max_3_	340–1000	3	0.9994	0.69	0.08	3.71
diclofenac	D1.1	ASA	max	350–760	2	0.9998	0.43	0.04	3.32
	D2.1		max_3_	340–700	3	0.9999	0.34	0.05	5.71
	D1.2	placebo	max	340–1500	3	0.9999	0.35	0.04	0.76
	D2.2		max_3_	340–700	2	0.9997	0.41	0.04	2.58
	D1.3	diclofenac	max	350–760	2	0.9998	0.43	0.05	1.49
	D2.3		max_3_	350–760	2	0.9998	0.43	0.05	1.49

**Table 4:** PLSR calibration and prediction model performance parameters for inline predictions of Table 2 containing coating.

Model	Normalization	Range [cm^−1^]	Factors	R^2^	RMSEC/%	SEC/%	RMSEP/% ASA	Rmsep/% Placebo	RMSEP/% Diclofenac
APD1	max	360–1400	2	0.9915	0.59	0.11	0.79	2.46	2.16
APD2	max_3_	380–1300	2	0.9925	0.83	0.14	0.59	2.31	2.31

**Table 5:** PLSR calibration and prediction model performance parameters for inline predictions of the TiO_2_-free coating.

Cores	Model	Prediction	Normalization	Range [cm^−1^]	Factors	R^2^	RMSEC/%	SEC/%	RMSEP/%
ASA	A3.3	ASA	max	1050–1460	4	0.9993	1.08	0.05	3.86
	A3.3	placebo	–						
	A3.2	diclofenac	–						
placebo	P3.1	ASA	max	770–1200	2	0.9992	0.91	0.10	2.65
	P3.2	placebo		340–1600	3	0.9993	0.83	0.09	1.16
	P3.3	diclofenac		850–1450	4	0.9999	0.06	0.06	2.71
diclofenac	D3.1	ASA	max	900–1250	2	0.9974	1.50	0.17	4.13
	D3.2	placebo		850–1200	3	0.9973	1.59	0.18	2.21
	D3.3	diclofenac		900–1560	4	0.9992	1.10	0.12	2.14
